# Supplementary figures and images for: The structural basis of the multi-step allosteric activation of Aurora B kinase
Source: eLife. 2023 May 25;12:e85328. doi: 10.7554/eLife.85328 (PMC10259393; doi:10.7554/eLife.85328)

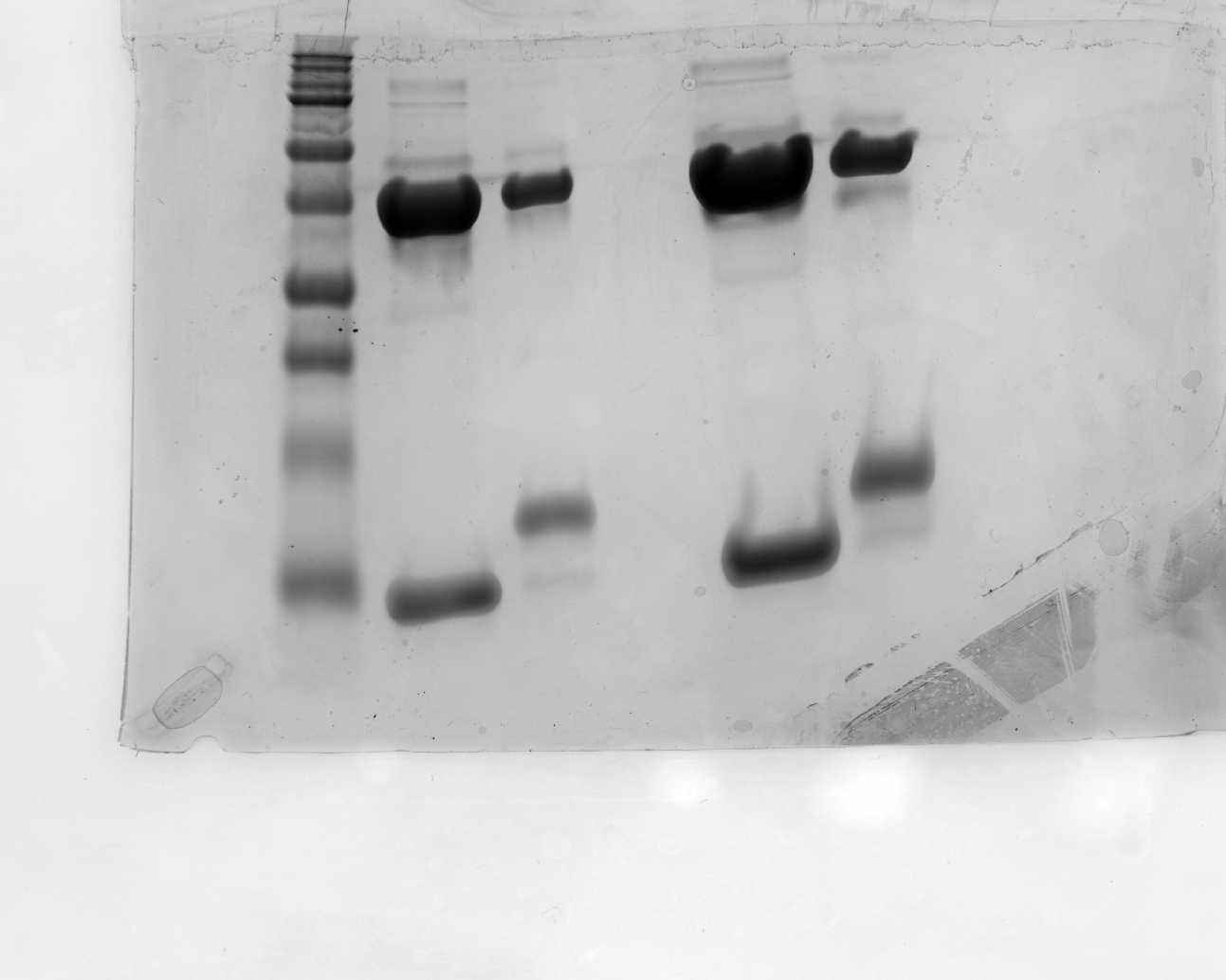

Supplement: Figure 3—figure supplement 1—source data 1. [file elife-85328-fig3-figsupp1-data1.zip › Figure-S7_source/Figure S7 gel2.tiff]

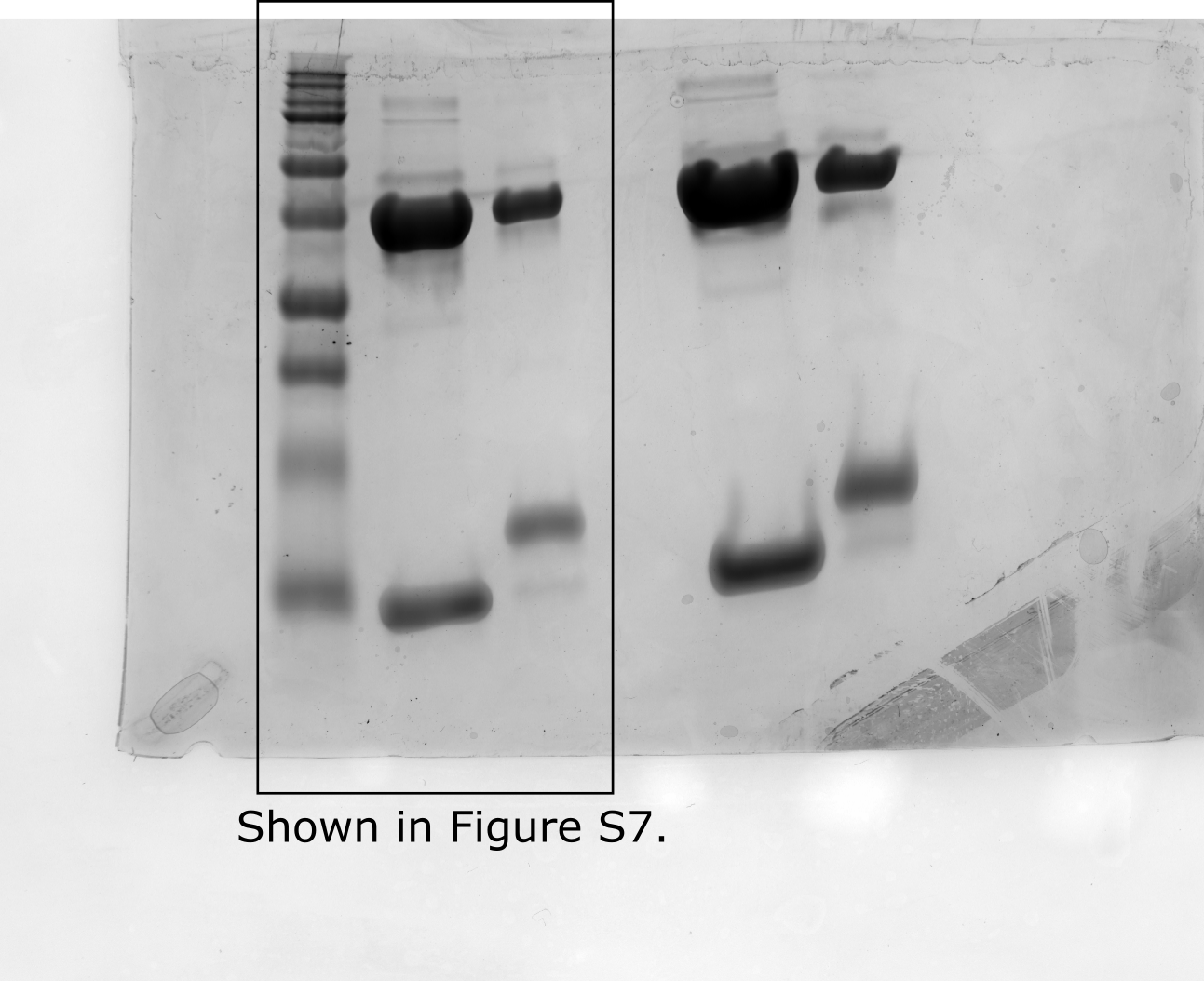

Supplement: Figure 3—figure supplement 1—source data 1. [file elife-85328-fig3-figsupp1-data1.zip › Figure-S7_source/Figure S7 gel2.png]

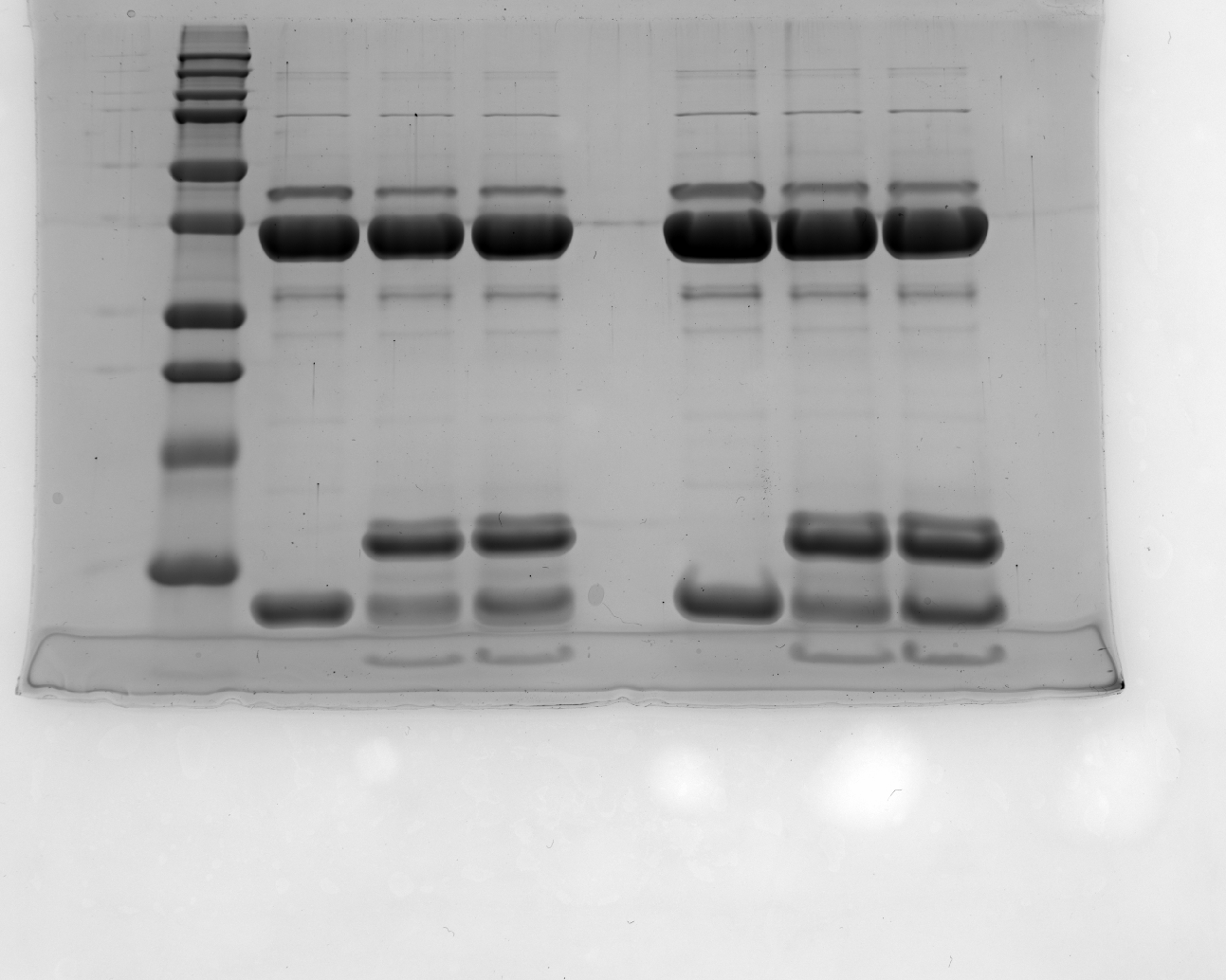

Supplement: Figure 3—figure supplement 1—source data 1. [file elife-85328-fig3-figsupp1-data1.zip › Figure-S7_source/Figure S7_gel1.tiff]

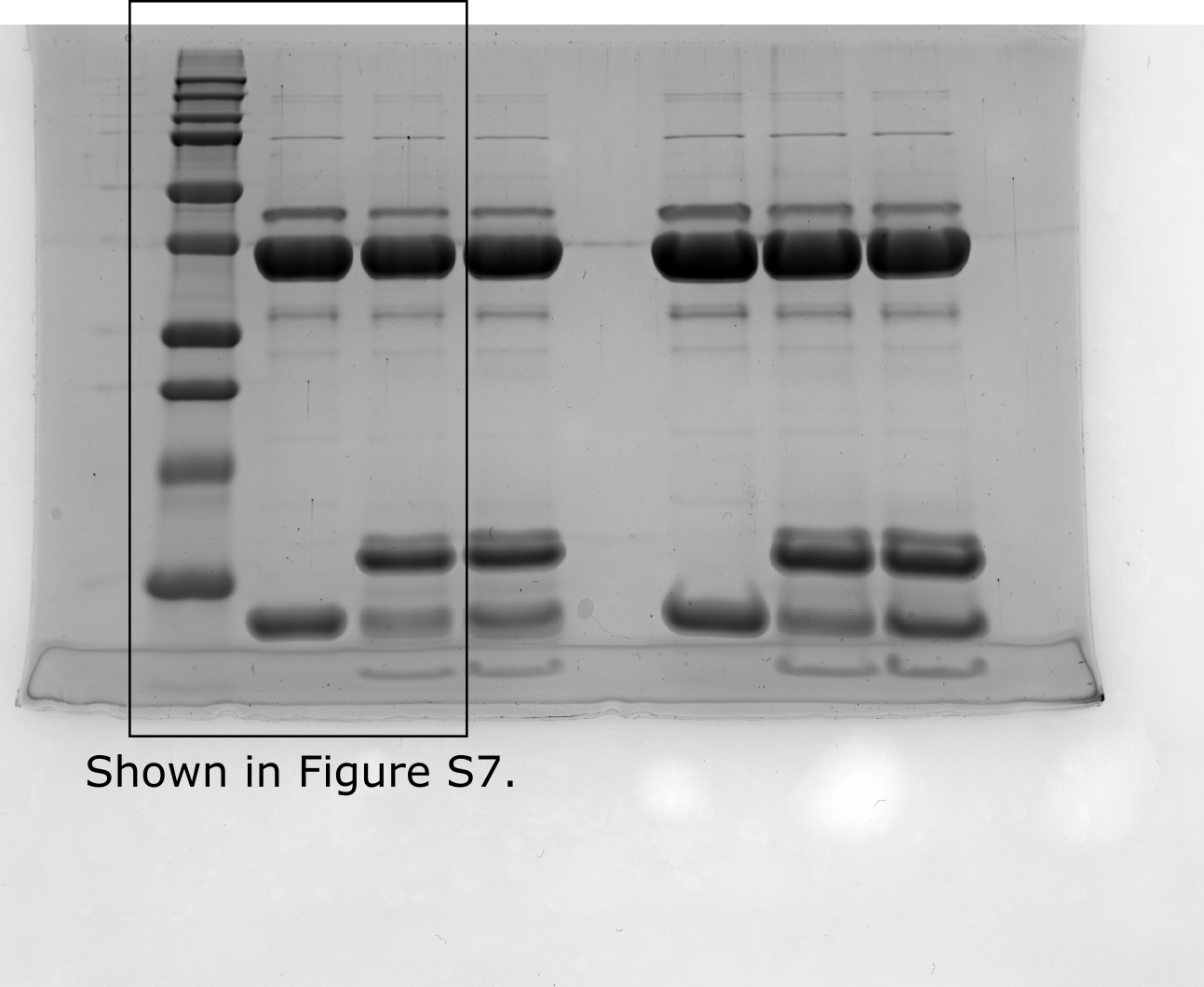

Supplement: Figure 3—figure supplement 1—source data 1. [file elife-85328-fig3-figsupp1-data1.zip › Figure-S7_source/Figure S7_gel1.png]

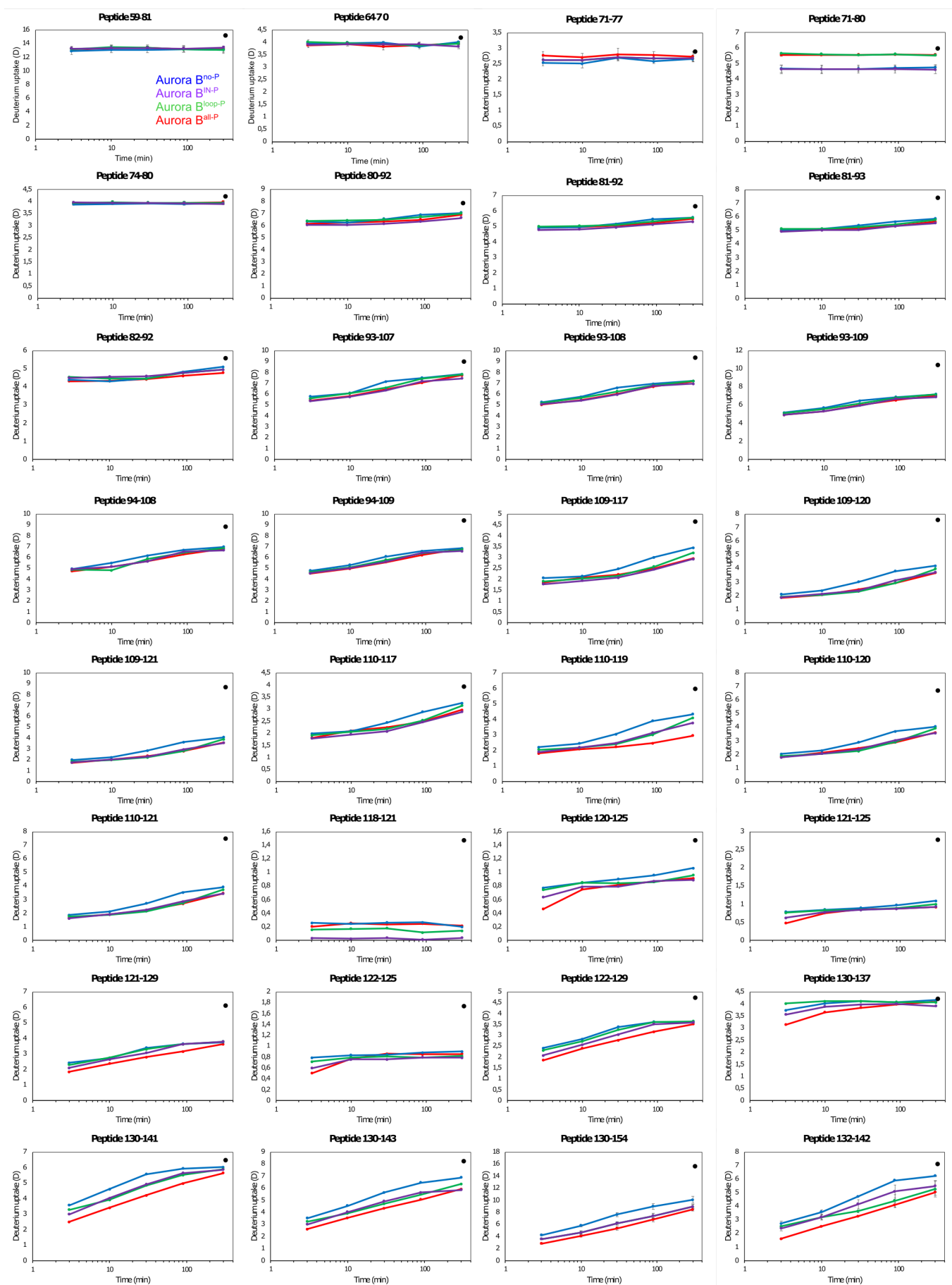

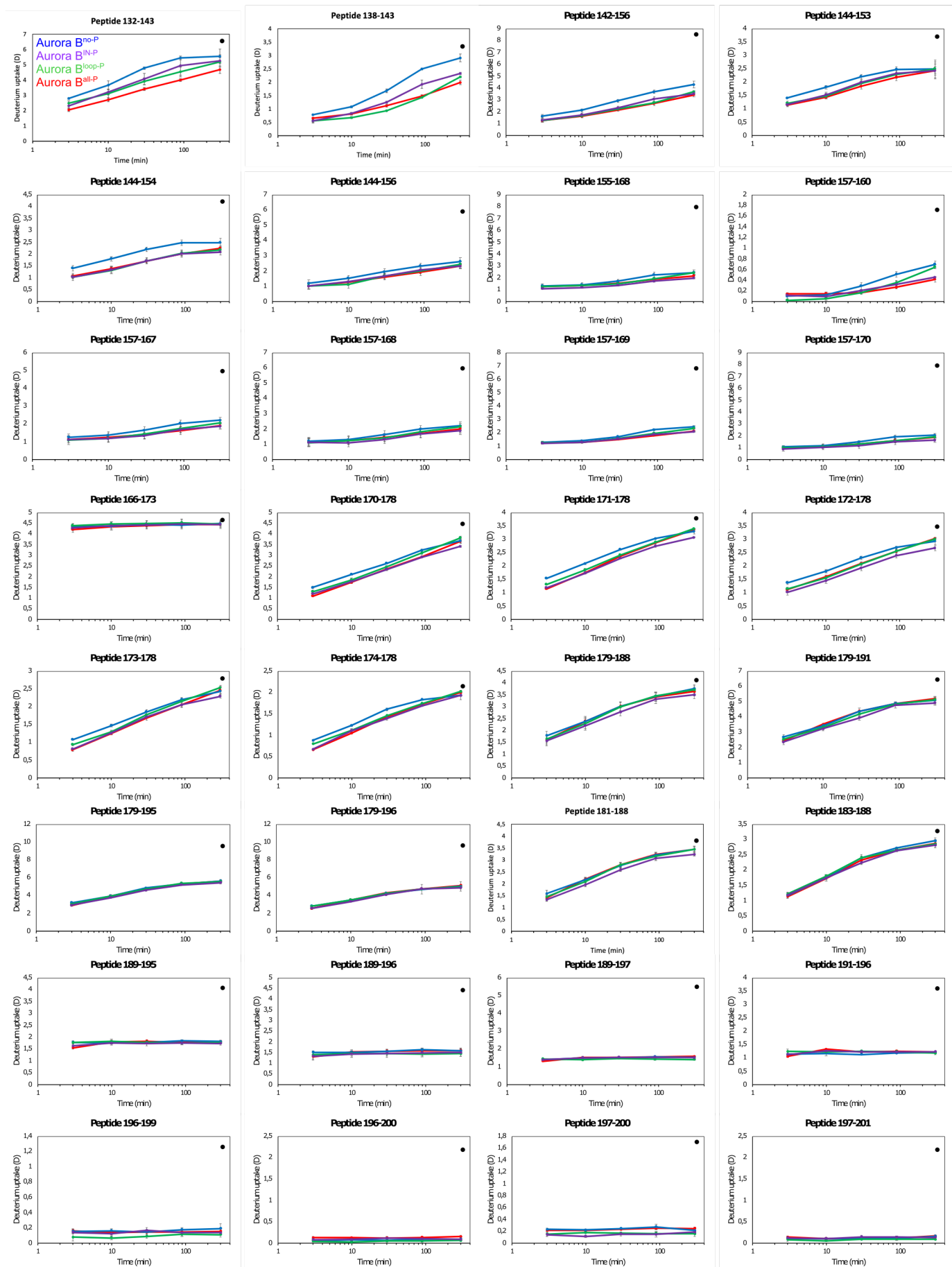

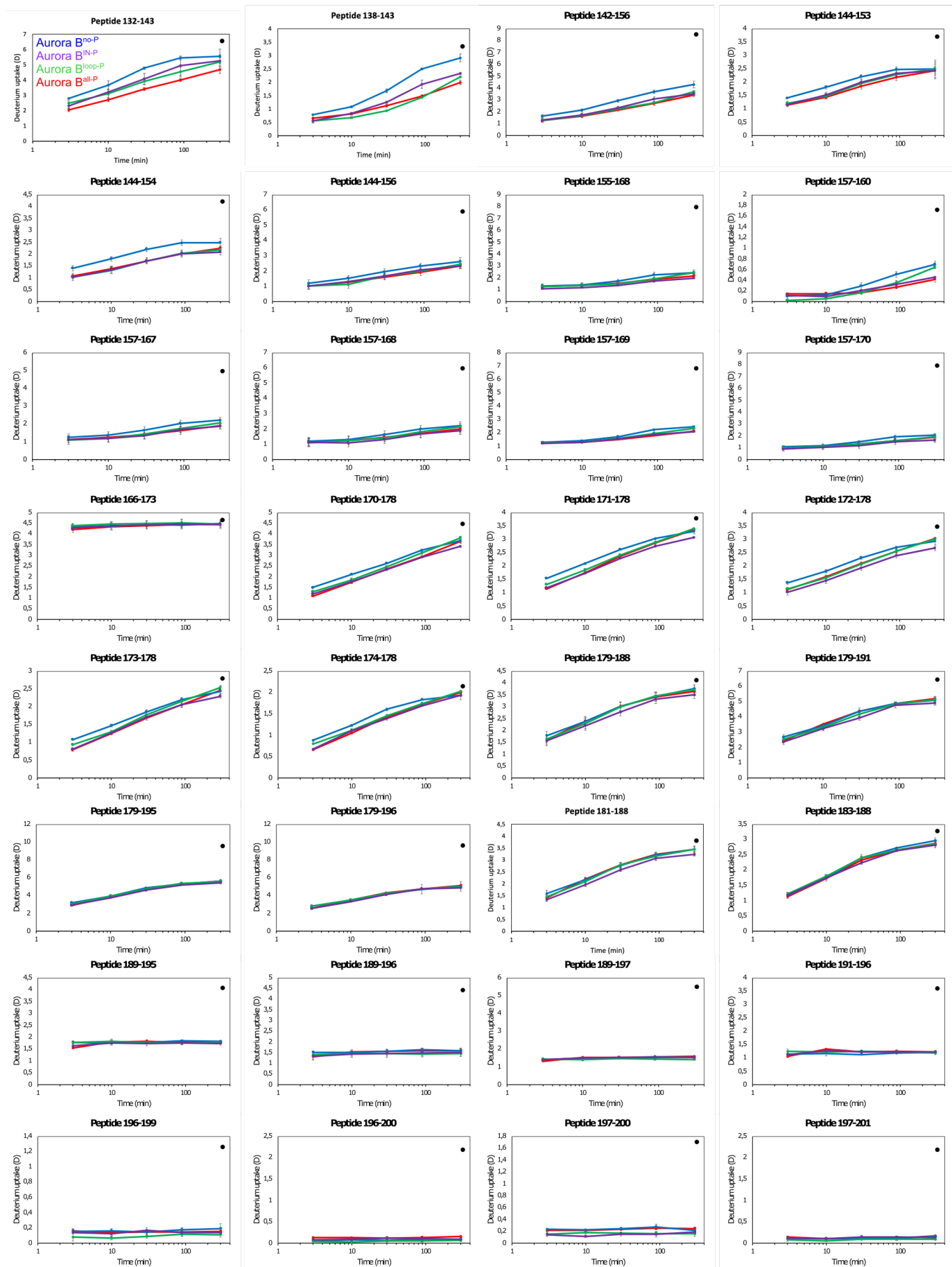

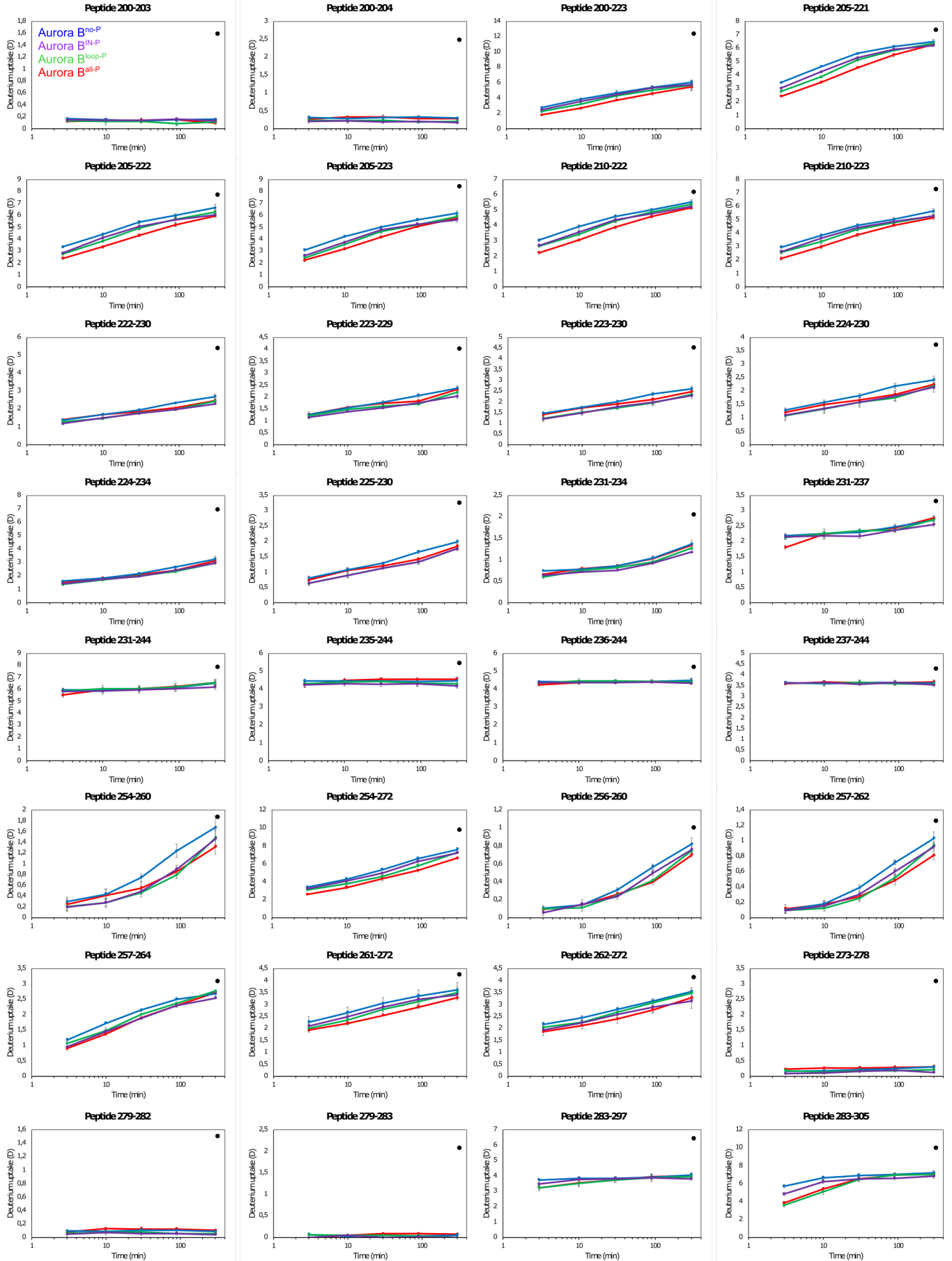

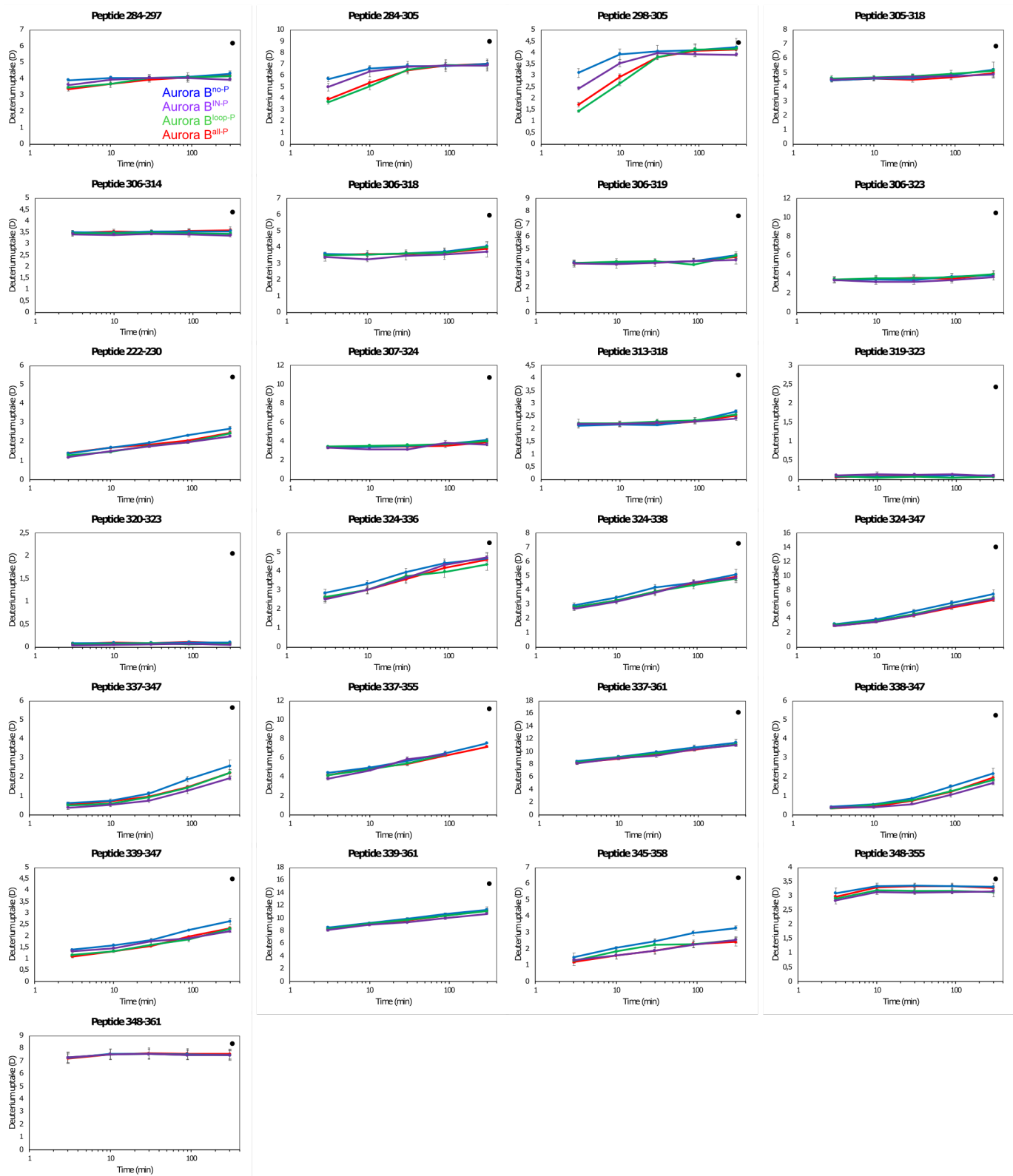

Supplement: Supplementary file 5. — All collected peptides are shown with the standard error calculated based on two replicates. The black dot in the corner of the graph indicates the exchange of the fully deuterated control for the peptide of interest. [file elife-85328-supp5.pdf]
